# Fast-growing epithelioid hemangioendothelioma of the liver: A case report

**DOI:** 10.1097/MD.0000000000022077

**Published:** 2020-09-04

**Authors:** Ye-hui Fan, He-nan Tang, Jian-peng Zhou, Qiang Fang, Shu-xuan Li, Kai Kou, Guo-yue Lv

**Affiliations:** The First Hospital of Jilin University, Changchun, Jilin, China.

**Keywords:** diagnosis, hepatectomy, hepatic epithelioid hemangioendothelioma, therapy

## Abstract

**Rationale:**

Hepatic epithelioid hemangioendothelioma (HEH) is a rare vascular tumor of the liver with malignant potential. It can be of solitary type, multifocal type, or diffuse type. Although there are some characteristic features on radiologic imaging, the definitive diagnosis of HEH is based on histopathology. The surgical treatment of HEH includes liver resection and transplant.

**Patient concerns:**

A middle-aged woman presented with easy fatiguability and anorexia for 1 month was found to have multifocal lesions on radiological imaging.

**Diagnosis:**

HEH was diagnosed by needle biopsy. It can be seen from imaging that this case is a multifocal form. The largest lesion increased from 3 to 3.3 cm within 2 months, with an increase of 9.45%; no other relevant literatures have been reported.

**Interventions:**

The possibility of liver transplantation was suggested to the patient. However, the patient refused transplantation and was successfully treated by radical right hepatectomy and resection of the left lobe lesion.

**Outcomes:**

She remained disease-free throughout a year follow-up period.

**Conclusion:**

HEH is a rare disease with characteristic radiological and pathological features. Although liver transplantation is the preferred treatment for multifocal HEH, surgical excision represents one alternative when the lesions can be guaranteed to be completely excised.

## 1 Introduction

Hepatic epithelioid hemangioendothelioma (HEH) is a rare vascular tumor that affects the liver. The condition has varying malignant potential and reported incidence of <0.1 per 1 lac population.^[1]^ Each HEH comprises of dendritic and endothelial cells. HEH is classified as solitary nodule, multifocal nodules, or diffuse type, based on the number of lesions.^[2]^ The solitary lesion type accounts for only 13% to 18% of all cases.^[1]^

Clinical manifestations of HEH include right hypochondriac pain, weight loss, anorexia, jaundice, and, in advanced cases, ascites. Approximately 25% of cases are asymptomatic.^[3]^ On contrast-enhanced magnetic resonance imaging (MRI), there are some typical features such as white or black “target-like” lesions, “lollipop” sign, capsular contraction, and submarginal distribution, as described by Gan et al.^[4]^ However, the diagnosis of HEH is based primarily on histopathology.

Curative treatment for HEH includes hepatectomy and liver transplantation. According to Blachar et al,^[2]^ resection is usually reserved for patients with solitary lesions. The rate of 5-year survival after resection is 75%.^[5]^ Liver transplantation is the treatment of choice for patients with multifocal lesions or the diffuse type of HEH.

Here, we report a case of a 57-year-old woman with multifocal HEH treated by liver resection after the patient refused liver transplantation.

## 2 Case presentation

A 57-year-old woman presented with easy fatiguability and anorexia for 1 month. The patient was referred to our hospital in April 2018. Physical examination showed vital signs within the normal range. The patient had a history of hypertension for 9 years. The patient's symptoms had resolved with amlodipine treatment. One year earlier, the patient had been diagnosed with hypothyroidism caused by 131I therapy for hyperthyroidism. The patient's hypothyroidism was controlled by thyroxine supplementation. She had undergone hysterectomy for uterine fibroids 9 years previously and cholecystectomy for gallstones 6 months before presentation in April 2018.

**Figure 1:**
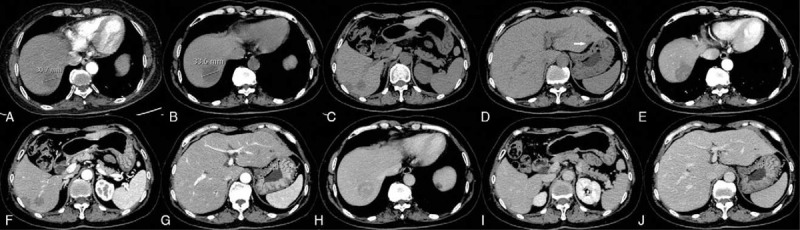
Computed tomography (CT). (A–C) Three rounded, hypodense lesions located in right liver lobe (diameter: 3 cm, 1 cm, 1.1 cm). (D) One oval-shaped hypodense lesion of 0.6 cm in the left lateral lobe of the liver. (E–G) Arterial-phase images showing patchy enhancement in the lesions and (H–J) hyperdense to isodense appearance in the portal phase. Please note the increase in size of the largest lesion from 30.7 mm (A) to 33.6 mm (B) in 2 months.

Routine blood tests were performed. The results of liver function tests were normal. Levels of CEA, CA 125, and CA 19-9 were normal. Computed tomography (CT) of the abdomen identified three hypodense circular lesions with diameter of 3, 1, and 1.1 cm, respectively, in the right liver lobe involving segments V, VI, and VII. CT also revealed 1 oval hypodense lesion of 0.6 cm in the left lateral lobe of the liver (segment III). All the lesions showed patchy enhancement during the arterial phase and homogenous enhancement during the portal phase (Fig. 1). With a provisional diagnosis of focal liver lesions of uncertain etiology, the patient was advised to undergo needle biopsy. However, the patient refused further treatment and was discharged for personal reasons.

**Figure 2:**
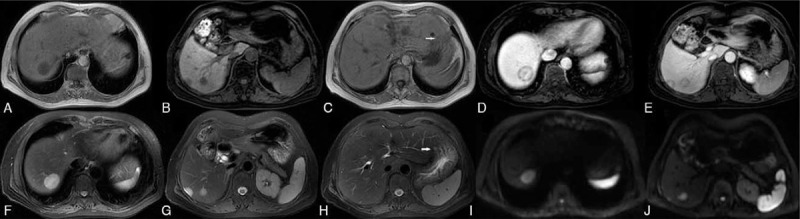
Magnetic resonance imaging (MRI). (A–C) Three hypointense lesions on T1-weighted images (T1-WI); (D,E) central enhancement with peripheral low-signal intensity after the administration of contrast (target-like lesions); (F–H) hyperintense signals on T2-weighted images (T2-WI); (I,J) mild high-signal intensity on diffusion-weighted images (DWI). The “Lollipop sign” (red circles) is a combination of 2 structures: the well-defined tumor and the adjacent occluded vein.

Two months later, the patient returned for treatment. Repeat CT revealed similar radiological findings, except that the largest mass in the liver had increased in diameter to 3.3 cm (Fig. 1). The results of MRI confirmed low-signal intensity on T1-weighted images (T1-WI) with central enhancement and peripheral low-signal intensity on contrast-enhanced imaging. T2-weighted images (T2-WI) revealed slight high-signal intensity; diffusion-weighted MRI (DWI) showed high signal intensity (Fig. 2).

**Figure 3:**
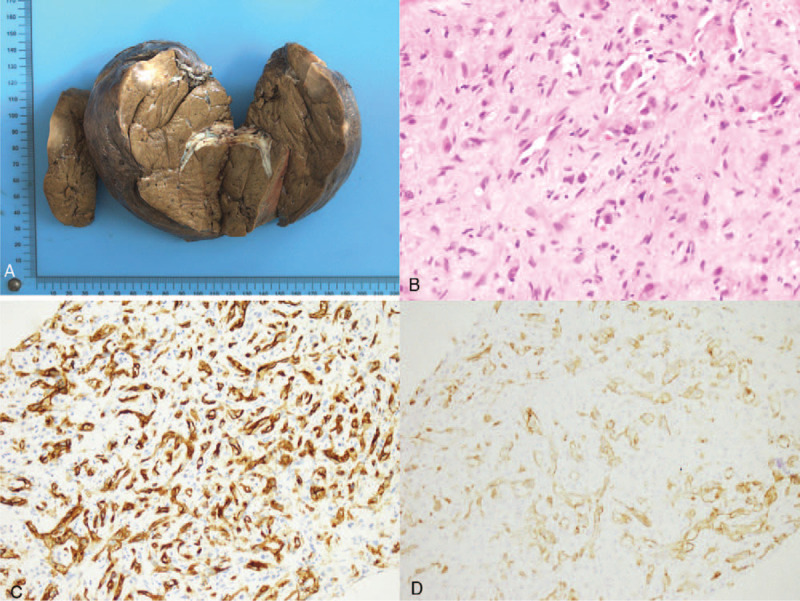
Pathology images: (A) gross appearance of the resected right liver lobe and partial left lateral liver lobe; (B) hematoxylin & eosin staining of the tumor, showing replacement of the sinusoids by vascular spaces; (C,D) positive immunohistochemical staining of the endothelium (CD-31, CD34).

The patient underwent needle biopsy of the right hepatic lesion. Hematoxylin & eosin staining revealed that the sinusoids had been replaced by vascular spaces. The results of immunohistochemistry (IHC) revealed endothelial cells stained positively for CD31 and CD34 (Fig. 3). Both sets of histopathological findings suggested HEH. The patient rejected the recommendation for liver transplantation because of financial reasons. The patient underwent open anatomical right hepatectomy and nonanatomical wide local excision of the left liver lobe lesion (Fig. 3). Lesions were identified intraoperatively using ultrasound. Intermittent portal clamping was performed during liver parenchymal transection, with total portal clamping time of 30 minutes. The operative time was 180 minutes, and the amount of intraoperative blood loss was 150 mL. No blood transfusion was required during the perioperative period. The postoperative course was uneventful, with hospital stay of 6 days.

At a year follow-up, the patient remained disease free. Postoperative pathology was consistent with the pathological diagnosis on needle biopsy.

## 3 Discussion

HEH is a rare hepatic tumor^[6]^ that affects women more commonly than men (male to female ratio, 1:1.5). Patients most commonly present with nonspecific clinical complaints related to right hypochondriac pain.^[7]^ Other reported symptoms include abdominal discomfort, anorexia, fever, vomiting, weight loss, ascites, and easy fatigability, as seen in the present case.^[3]^ The exact cause of HEH is not known. Previously reported cases have been found to be associated with various factors such as exposure to asbestos, vinyl chloride, oral contraceptives, Crohn disease, alcohol consumption, hepatitis B and C, liver trauma, and sarcoidosis.^[3]^ The patient presented above had surgical history of file://C:\Program Files (x86)\Youdao\Dict\7.3.0.0817\resultui\dict\?keyword=cholecystectomy, which may have led to minor liver trauma. However, future studies are needed to confirm these associations.

The differential diagnosis for HEH includes cavernous hemangioma, multifocal hepatocellular carcinoma, peripheral cholangiocarcinoma, and metastatic lesions. However, there are some specific imaging features on CT and MRI that should trigger high suspicion for HEH. The tumor appears as a well-defined hypodense lesion on CT scans. Compared with the adjoining normal hepatic parenchyma, HEH appears as hypointense on T1-WI MRI and heterogeneously hyperintense on noncontrast T2-WI MRI. HEH has central hyperintense signals with a high-signal intensity halo on DWI; this appearance is known as a “target-like” lesion and is present in 61% of cases.^[2]^ Target-like lesions were observed in the present case.

The enhancement features depend upon the pattern of blood supply on contrast-enhanced MRI. Multifocal HEH lesions display 4 types of enhancement patterns on MRI: homogeneous enhancement with mild irregularity; central hypointensity with peripheral enhancement in the arterial phase, with central hyperintensity with a thin hypointense ring during the portal venous and delay phases (black “target-like” sign); central nodular enhancement in the arterial phase and ring-like enhancement in the portal venous and delay phases (white “target-like” sign); and peripheral nodular enhancement in the arterial phase with centripetal enhancement in the portal venous and delay phases.^[4]^ Another typical feature of HEH is “lollipop” sign, in which the hypointense hepatic or portal vein or their branches lying perpendicular to the lesion and terminating at its edge give the appearance of a lollipop.^[8]^ The index case showed this feature on MRI. Some reports mention capsular retraction to be an important sign of HEH, but it is most common in lesions > 2.0 cm.^[9]^^18^F-FDG PET/CT can be used to detect potential metastases in HEH patients, which typically show mild to moderate FDG uptake.^[10]^

Currently, the definitive diagnosis of HEH is based on histopathology. These tumors range from paucicellular to moderately cellular. They are composed of spindle cells and epithelial cells, seen singly or arranged in clusters.^[11]^ HEH stains positively for endothelial markers on IHC: 100% of tumors are positive for factor VIII-related antigen, 94% for CD34, and 86% for CD31.^[1,6]^ Our case showed positive staining for CD31 and CD34. Recently, Kelleher et al^[12]^ found that podoplanin (D2-40) staining can also be used to differentiate HEH from other vascular lesions.

There is no widely accepted treatment strategy for HEH because of its heterogeneous status and association with variable clinical outcomes.^[13]^ Some experts advocate surgical excision in patients with a single or few lesions. Liver transplantation is usually reserved for patients with extensive or multiple lesions, in the absence of distant metastasis.^[14]^ After refusing liver transplantation due to financial concerns, the patient elected to undergo radical right hepatectomy with resection of the left lobe lesion.

Among the various therapeutic methods reported from 1984 to 2005, the surgery considered as gold-standard treatment for HEH is liver transplantation (44.8%).^[3]^ Mehrabi et al^[5]^ reported outcomes in 110 patients with HEH who underwent liver transplantation during the period from 1987 to 2005. The rate of 5-year survival was 64%, with 11% of patients dying of recurrent HEHE before that time-point. Overall disease-free survival ranges from 4 months to 10 years (mean, 59 months).^[1]^ Mehrabi et al^[15]^ reported that liver transplantation yields good results in HEH, even in cases with extrahepatic disease and lymph nodal involvement. However, approximately 25% of patients develop recurrence after liver transplantation by 49 months postoperatively.^[16]^ Significant risk factors for recurrence include macrovascular invasion, pre-liver transplantation waiting time ≤120 days, and hilar lymph node invasion.^[17]^ Tan et al^[17]^ believe that adjuvant chemotherapy may be useful for the prevention of recurrence in patients with extrahepatic disease.^[18]^

Various chemotherapeutic drugs (including doxorubicin, 5-fluorouracil, vincristine, thalidomide, interferon-α, and monoclonal antibodies against vascular endothelial growth factor) have been used for the treatment of HEH.^[19]^ Soape et al^[20]^ reported a case of HEH with good response to sorafenib monotherapy. In selected cases, radiotherapy may be used for patients unfit for surgery or chemotherapy.

Other literatures mostly focus on tumor size and quantity,^[6]^ as most authors have to make a decision on how to deal with the lesion at once and have no chance to notice the tumor growth rate. This literature provides a reference on the growth rate of HEH, increasing in diameter by 9.45% within 2 months, faster than benign tumor of liver with little change in several months, which provides evidence for the study of the biological characteristics of HEH, and provides a reference for the selection of its operation time.

HEH is a rare disease that should be suspected in patients with multiple liver lesions with specific imaging features on CT and MRI. However, the definitive diagnosis of HEH can only be made on histopathology. Liver transplantation and surgical excision are the main curative treatment options for HEH.

## Author contributions

**Conceptualization:** Kai Kou, Guo-yue Lv.

**Data curation:** Ye-hui Fan, He-nan Tang.

**Investigation:** Jian-peng Zhou, Qiang Fang.

**Methodology:** Shu-xuan Li.

**Project administration:** Kai Kou.

**Supervision:** Guo-yue Lv.

**Writing – original draft:** Ye-hui Fan, He-nan Tang.

**Writing – review & editing:** Kai Kou, Guo-yue Lv.
